# Supplementary material for: A high-resolution mRNA expression time course of embryonic development in zebrafish
Source: eLife. 2017 Nov 16;6:e30860. doi: 10.7554/eLife.30860 (PMC5690287; doi:10.7554/eLife.30860)
Supplement: Supplementary file 6. [file elife-30860-supp6.zip › biolayout-clusters-files/Cluster251-genes.html]

Cluster251


# Cluster251: Genes

| Ensembl ID | Gene Name | Chr | Start | End | Biotype |
| --- | --- | --- | --- | --- | --- |
| ENSDARG00000059908 | CCDC86 | 1 | 51817272 | 51826584 | protein\_coding |
| ENSDARG00000017696 | diexf | 13 | 31794804 | 31808062 | protein\_coding |
| ENSDARG00000054259 | nat10 | 18 | 38235802 | 38263863 | protein\_coding |
| ENSDARG00000038027 | nol12 | 3 | 1381699 | 1387384 | protein\_coding |
| ENSDARG00000079973 | pus10 | 1 | 53081892 | 53097694 | protein\_coding |
| ENSDARG00000032516 | rrp36 | 22 | 15317570 | 15326163 | protein\_coding |
